# CYGNSS data map flood inundation during the 2017 Atlantic hurricane season

**DOI:** 10.1038/s41598-018-27673-x

**Published:** 2018-06-19

**Authors:** Clara Chew, John T. Reager, Eric Small

**Affiliations:** 1 University Corporation for Atmospheric Research, Boulder, CO USA; 2 Jet Propulsion Laboratory, California Institute of Technology, Pasadena, CA USA; 3 University of Colorado Boulder, Boulder, CO USA

## Abstract

The 2017 Atlantic Hurricane Season was one of the most active and destructive on record, leading to significant flooding in many parts of the United States and the Caribbean. During flooding events such as these, there is an urgent need to quickly map in detail which areas have been severely affected, yet current satellite missions are not capable of sampling the global land surface at high enough spatio-temporal scales for flooding applications. Here, we demonstrate a novel approach to high-resolution flood mapping by repurposing data from the new NASA mission, CYGNSS. The CYGNSS multi-satellite constellation was designed for frequent temporal sampling of the ocean surface in the tropics. We demonstrate that CYGNSS data provide clear signals of surface saturation and inundation extent over land at higher spatio-temporal resolution than radiometers like SMAP. Using a simple thresholding technique, we are able to estimate that approximately 32,580 km^2^ of land area in Texas flooded during Hurricane Harvey, and approximately 7210 km^2^ of land area flooded in Cuba during Hurricane Irma, or about 7% of Cuba’s total area.

## Introduction

The timely mapping of surface water during flooding events is difficult due to the highly dynamic nature of inundation^1^. As soils saturate and swell with rain, floodwaters can rise and fall within a matter of days, and flooding extent is often spatially discontinuous depending on the terrain^2,3^. Yet detailed mapping is important, as flooding is one of the most destructive natural disasters and poses significant risks to human life^4–6^. The ability to rapidly map the areas most impacted by flooding events can help first responders to find impacted regions, help scientists better understand the temporal and spatial evolution of these types of disasters, and can create a reference system of places that may be affected again in the future^7^. Mapping approaches based on satellite data have created the opportunity to cover large areas in a relatively short time frame^8^.

Despite the critical need to map flooding events, current space-based mapping techniques still fall short in key areas^9^. Optical remote sensing often fails to map floods as they are happening, as the Earth’s surface is usually obscured by clouds during flooding events^10^. Microwave remote sensing is generally accepted as the best way to map surface water, as it can penetrate clouds and, to some extent, vegetation^11,12^. However, existing satellites either provide data with too coarse of a spatial footprint (~40 km, radiometers) to provide information pinpointing the most heavily affected areas, or data with fine spatial resolution (10s m – few km, monostatic radars) but with temporal repeat cycles so long (>1 week) that they are not used operationally during floods, nor can scientists be sure the sensor was able to capture the extent of maximum inundation^1,13,14^. Distribution of data to the public domain from monostatic radars is also often severely restricted.

Here, we present evidence that surface-reflected Global Navigation Satellite System (GNSS) signals captured high-resolution flood inundation dynamics during the 2017 hurricane season for the southeastern United States and Caribbean. The information contained in these L-band bistatic radar signals could provide a compromise between that provided by traditional microwave remote sensing satellites. For instance, the spatial resolution of the data explored here is on the order of a few kilometers, with a temporal repeat period of 1–3 days. The spatial and temporal resolution of surface-reflected GNSS signals approaches that required to quantify flood inundation dynamics at appropriate scales. Using these signals, we quantify the timing of peak inundation extent, the duration of flooding, and map maximum inundation extent for Texas and Cuba during Hurricanes Harvey and Irma.

### Surface-reflected GNSS signals

This analysis repurposes data collected by the Cyclone GNSS (CYGNSS) constellation in order to examine flood inundation. GNSS is a generic name for the satellite-based navigation systems, including the well-known Global Positioning System (GPS), that provide precise geo-location information for a receiver at any location on the Earth’s surface. CYGNSS is a NASA mission that launched in 2016 consisting of a constellation of eight small satellites carrying GNSS receivers specially designed to capture surface-reflected GNSS signals (called ‘signals of opportunity’) over the tropics to retrieve ocean surface wind speed^15–17^. Because CYGNSS is a constellation, the eight satellites have a much more frequent orbital repeat than a single wide-swath satellite, making them ideal for high temporal resolution observations. The receivers also collect data over the land surface, but these data are not used in the retrieval products because they are not within the designed target observational area. Although CYGNSS was not designed to map flooding, here we will show that the surface reflectivity (SR) data are particularly sensitive to changes in inundation extent and use these data to map surface flooding during the 2017 hurricane season.

It has long been known that L-band signals such as those from GNSS are sensitive to the amount of water or moisture content on the ground, with increased moisture content or surface water increasing the reflectivity of the surface^18^. Given that an increase (decrease) in SR is known to be related to an increase (decrease) of soil saturation or surface water^18–23^, here we equate changes in SR to changes in inundation extent, with larger changes in SR indicative of larger changes in inundation. Currently, there is no retrieval algorithm to equate the specific changes of SR recorded by spaceborne GNSS-R receivers to maps of surface inundation. Increases in SR could indicate either an increase in soil saturation or an increase in inundation or surface ponding, though we assume that more significant increases in SR are indicative of more water, either in the surface soil layer or as ponded water. Details on the exact approach are provided in the methods section.

In order to quantify timing and duration of flooding, we analyze temporal changes in SR relative to their mean pre-hurricane levels. When SR anomalies have reached their maximum, inundation extent has peaked, and when SR anomalies have returned to pre-hurricane levels, we assume that flooding has receded, and the soil is no longer saturated. In order to map inundation extent, we choose a SR threshold to identify flooded versus non-flooded areas. Thresholding is a common approach used to map inundation with monostatic radar observations, though in these systems inundated areas are indicated by a lack of signal return, which is the opposite case to our bistatic radar observations^14,24^. We confirm our methodology using both optical and microwave data.

### Flood Inundation in Texas

**Figure 1:**
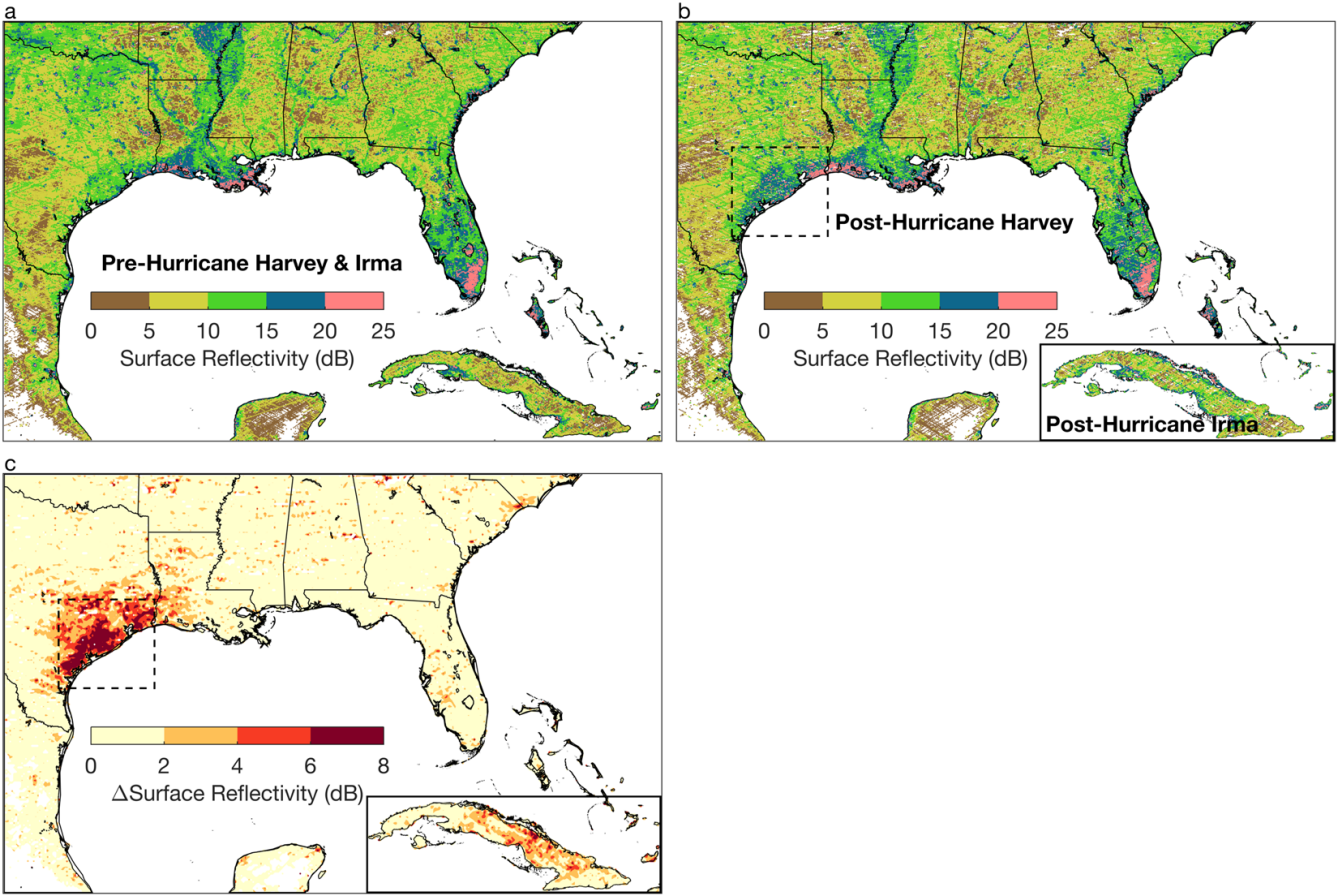
Observations of surface reflectivity from CYGNSS over the southeastern United States and Caribbean. (**a**) Surface reflectivity observations for the time period Jul 1–Aug 20, 2017, before the hurricane season began. (**b**) Surface reflectivity observations after Hurricane Harvey (Aug 25–Sep 15, 2017) for the southeastern United States. Observations in the inset of Cuba were recorded in the time period after Hurricane Irma (Sep 8–Sep 30, 2017). (**c**) Observed change in surface reflectivity after Hurricanes Harvey (southeastern United States) and Irma (Cuba inset). All figures made with MATLAB R2016b.

Figure 1a shows SR observations recorded by CYGNSS in the southeastern United States and Caribbean prior to the start of the 2017 hurricane season. Higher SR is observed in wetland and riverine areas along the Mississippi River Basin and Delta, the Everglades in southern Florida, and along the coasts of Georgia and South Carolina. Lower SR is observed both in drier areas as well as areas with dense forest cover and mountainous terrain—like all radar measurements, surface-reflected GNSS signals are also affected by rough terrain.

**Figure 2:**
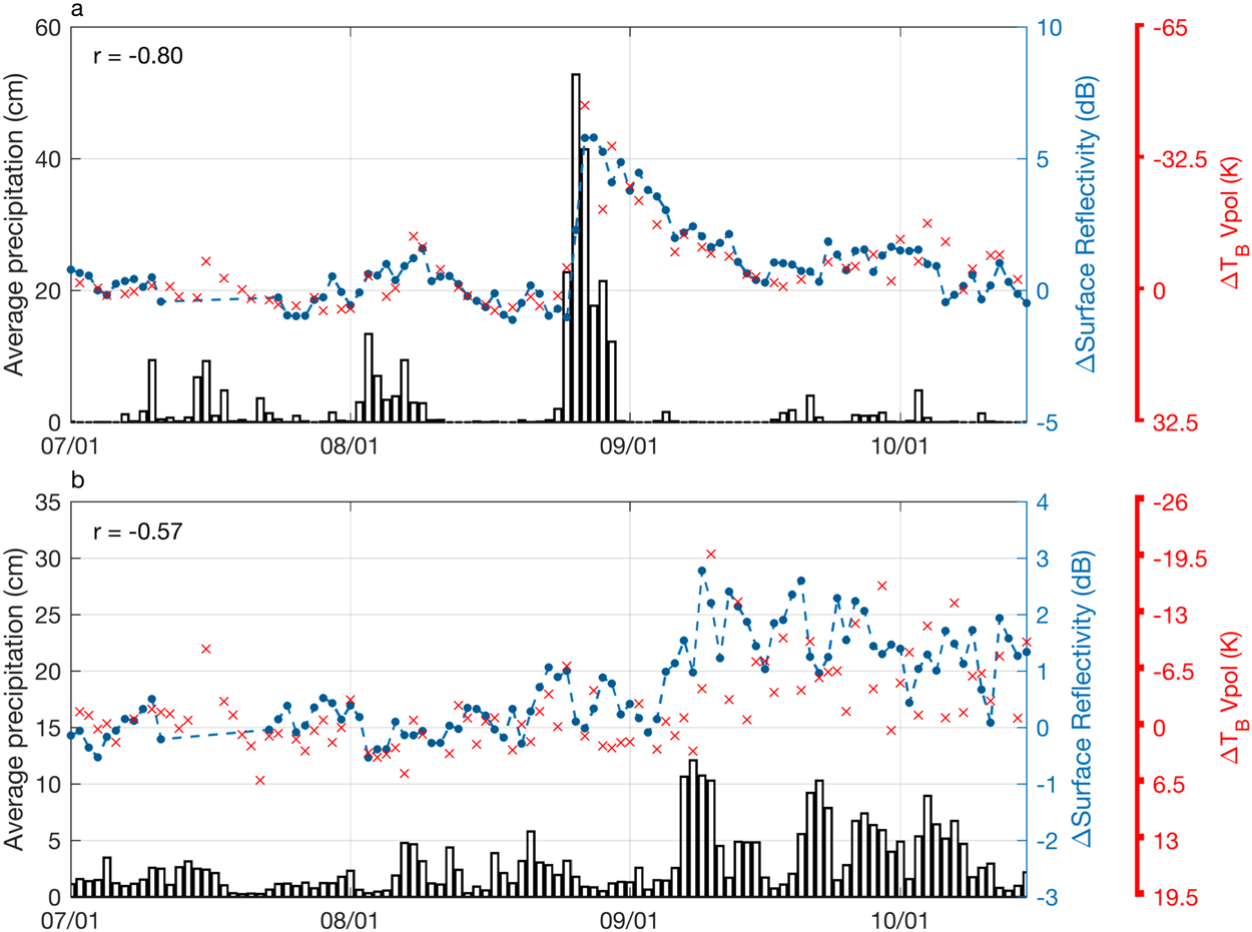
Changes in the daily mean observations of surface reflectivity (blue dots) for the Texas coastline (**a**) and Cuba (**b**) outlined by the black boxes in Fig. 1 for 2017. Also shown are mean precipitation^26^ (bars) and observations of daily mean changes in radiometric brightness temperature from SMAP^27^ (red ‘x’s) for the same regions. Because of ocean contamination in the SMAP coarse resolution data, there is more noise in the observations over Cuba and disagreement with the SR observations (r = −0.57).

After Hurricane Harvey, SR increased most significantly along the Texas coastline (Fig. 1b,c). A time series of mean changes in SR in Texas (for the region outlined by the black box in Fig. 1**)**, relative to observations recorded prior to the hurricane, are shown in Fig. 2a. Changes in SR along the Texas coastline are greatest in the time period during and directly following Hurricane Harvey. Larger changes in SR are indicative of more extensive flooding. SR in Texas decreases following Hurricane Harvey, indicating flooding recession, though values are still elevated through the first two weeks of September (Fig. 2a). Plotted alongside SR observations are corresponding observations of changes in L-band radiometric brightness temperature for the same region, which were recorded by NASA’s Soil Moisture Active Passive (SMAP) satellite. SMAP has produced similar maps of flooding in Texas during Hurricane Harvey^25^. Given that the temporal evolution of the SR observations mimics that of the brightness temperature observations (r = −0.80), we can be confident that changes in SR are indicative of changes in surface inundation.

**Figure 3:**
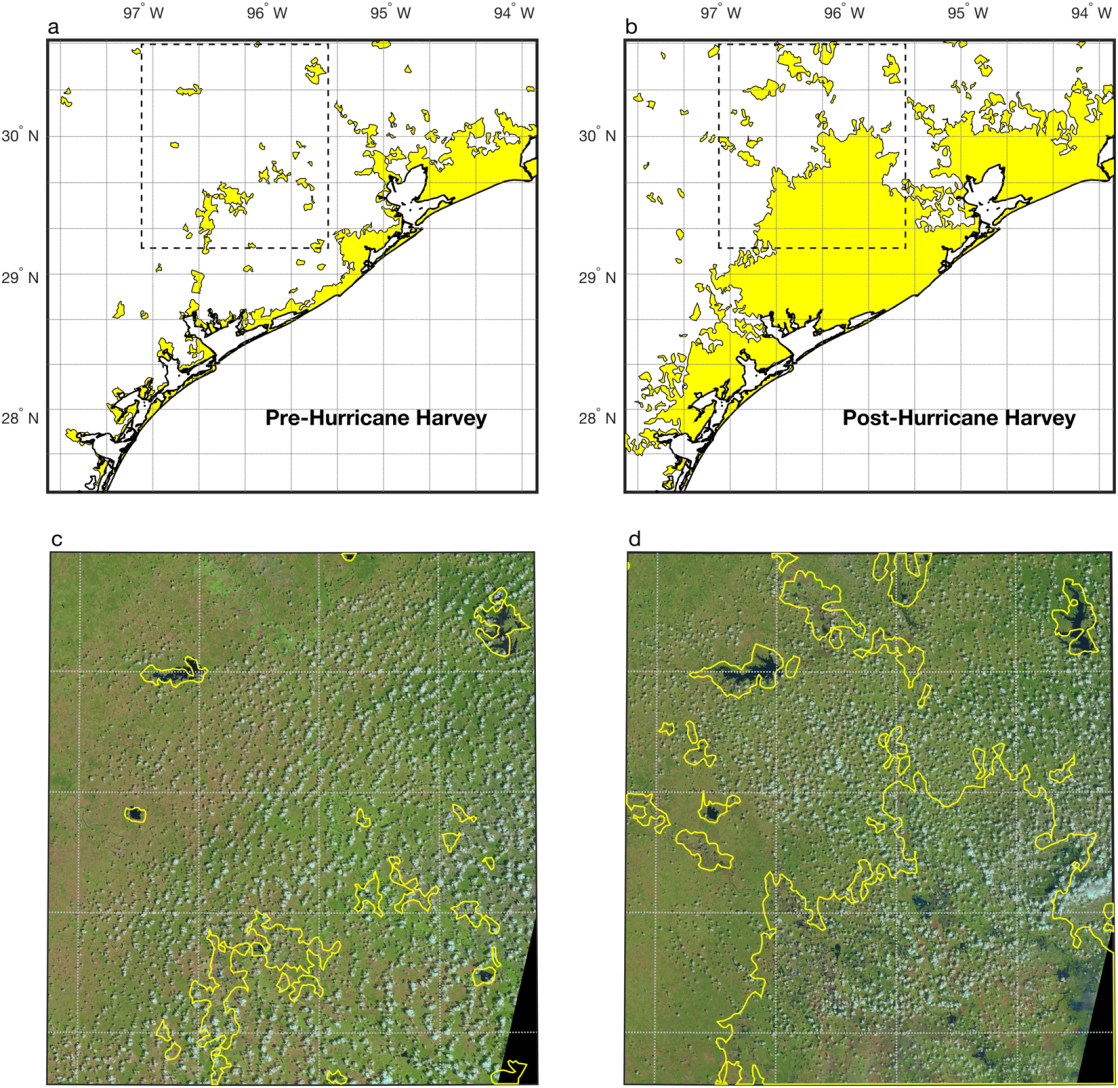
Maps of saturated and inundated areas in Texas before (**a**) and after (**b**) Hurricane Harvey (yellow filled regions). Yellow contours in (**c**) and (**d**) are the same data, overlaid on optical images from Landsat before^28^ (Aug 19, 2017) and after^29^ (Sept 4, 2017) Hurricane Harvey, for the black dashed boxes in (**a**) and (**b**). The grey grid-lines represent the SMAP mission spatial resolution.

Maps of inundation for the part of Texas most heavily impacted by Hurricane Harvey are shown in Fig. 3 for both before (Fig. 3a) and after (Fig. 3b) the hurricane. Regions in yellow outline inundated areas, which significantly expanded in the aftermath of the hurricane. Figure 3c,d show inundated areas for a selected part of Texas (black dashed boxes in Fig. 3a,b) overlaid on top of optical images. The images were taken before (Fig. 3c) and after (Fig. 3d) Hurricane Harvey, though the image after Hurricane Harvey was taken on September 4, 2017, several days after peak inundation extent.

SR observations from Fig. 2a indicate that flood extent peaked in Texas on August 28, which was three days after Hurricane Harvey made landfall and two days after peak precipitation. SR observations indicate that flooding from Hurricane Harvey in Texas had all but receded by September 15, over two weeks from the time when flood waters initially began to rise. The expansion of inundated area shown in Fig. 3 suggest that, at peak flooding, inundation extent had expanded by 32,580 km^2^ in this region due to Hurricane Harvey.

### Flood inundation in Cuba

Cuba also experienced significant flooding during the 2017 hurricane season, most significantly during and in the aftermath of Hurricane Irma. Figure 1a,b shows SR observations recorded before and after Hurricane Irma, with Fig. 1c showing the change in observed SR. Figure 2a shows a time series of daily-averaged changes in SR for Cuba and surrounding islands, relative to pre-hurricane values, alongside precipitation and radiometric brightness temperature observations. Like Texas, changes in SR mimic changes in radiometric brightness temperatures, though there is more noise in both SR and brightness temperature observations. The SR time series indicates that flooding peaked one day after peak precipitation, on September 9, 2017. Unlike Texas, SR remained elevated well into October and November (not shown), probably due to the continued rainfall.

**Figure 4:**
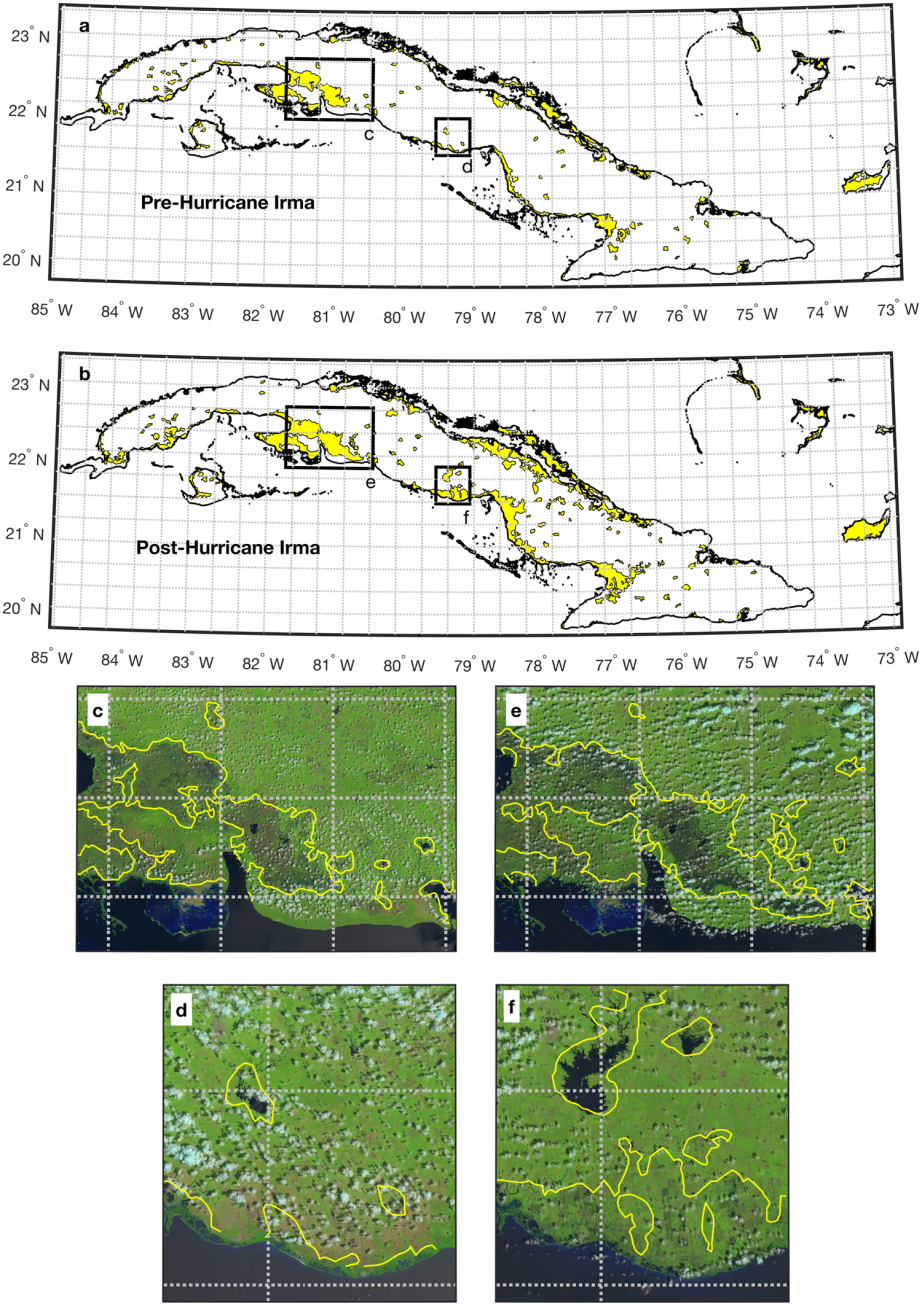
Map of inundated areas in Cuba before (**a**) and after (**b**) Hurricane Irma (yellow filled contours). (**c** and **d**) are insets of the black outlined boxes in (**a**) overlaid on optical images from Landsat before^30^ (Sept 7, 2017) and after^31^ (Oct 9, 2017) Hurricane Irma. (**e** and **f**) are insets of the black outlined boxes in (**b**) overlaid on Landsat images before^32^ (Aug 31, 2017) and after^33^ (Oct 2, 2017) Hurricane Irma. Grey dashed boxes are spatial footprints of the SMAP satellite.

Figure 4 shows maps of inundation for Cuba and part of the West Indies before and after Hurricane Irma. Before Hurricane Irma, wetland areas, lakes, and reservoirs are identified by high SR values. After Hurricane Irma, regions of high SR had expanded in area, particularly along the coasts. To confirm that these data are indeed indicating an increase in flooding in these areas, we compared SR observations to optical images taken in the weeks before and after Hurricane Irma for regions outlined by boxes c-f. The expansion of inundation extent before and after Hurricane Irma is evident in both optical images and SR contours. These inundation maps indicate that approximately 7210 km^2^ flooded in mainland Cuba during Hurricane Irma, which is ~7% of Cuba’s total area.

### Applications potential for inundation signals from GNSS

Despite the fact that the CYGNSS satellites were not designed to monitor land surface flooding, the data these satellites provide demonstrate capabilities similar to those of state-of-the-art radiometers and monostatic radars in terms of sensitivity to soil wetness and inundation. CYGNSS also shows improved potential for higher spatio-temporal resolution due to high repeat frequency.

To highlight the improved spatial resolution of CYGNSS data relative to satellite radiometers, gridded dashed boxes in Figs 3 and 4 are outlines of the spatial footprints of the SMAP satellite. This and other satellite radiometers are not able to resolve the sub-footprint scale changes in inundation, nor can they provide reliable data along the coasts, due to contamination by the ocean signal^27,34^. In Cuba, only a small fraction of the SMAP grid cells do not overlap with the coastal regions, which were the areas that were most severely impacted by Hurricane Irma. Conversely, the theoretical spatial footprint for a GNSS reflection recorded by a satellite at the altitude of CYGNSS is on the order of 0.5 × 0.5 km^2^, if the surface is relatively flat, as is the case of inland water^35^. Data processing performed by CYGNSS degrades the spatial resolution, such that the smallest resolution is approximately 7 × 0.5 km. Surface roughness introduced by overlying vegetation will also degrade the surface resolution, though at this time bistatic scattering models are not mature enough to indicate the extent of this degradation. Regardless, observational evidence presented here (e.g., Figs 3 and 4) and elsewhere^36–38^ show that the typical spatial resolution of reflections recorded by CYGNSS are indeed finer than that of radiometers.

Calculations of the temporal repeat frequency of CYGNSS data depend on the assumed footprint size. For example, the CYGNSS mission estimates that the mean revisit time over the ocean, where the spatial footprint is ~25 × 25 km, is approximately seven hours^17^. Due to the finer spatial resolution over the land surface, the temporal repeat time is longer. However, if one were to assume that the spatial footprint is ~9 × 9 km, then on average a third of the tropics would be sampled every day, given a statistical repeat time of ~3 days.

One of the defining characteristics of surface-reflected GNSS signals is their ability to map small- to moderate-scale changes in inundation extent. This, combined with their relatively high temporal repeatability, has important implications for water managers and disaster management teams. Although CYGNSS was not designed to measure land surface conditions, several options present themselves from our results.

First, the current data set resulting from CYGNSS can be processed with the goal of land surface observation, which would require new algorithm development and testing. Because of the loss of NASA SMAP’s active radar instrument, there is a gap in the earth observing system for high-resolution soil moisture and surface inundation observations. CYGNSS data could fill that gap quickly. As algorithms are developed and new datasets become available, CYGNSS data will then be useful for applications in the flood and emergency management communities.

Second, future GNSS satellite receiver constellations, as well as other signal of opportunity missions, could be optimized for land surface remote sensing and provide data with an even better temporal repeat time to retrieve sub-daily observations. Because the cost of small satellites is generally cheaper than large satellite platforms, and constellations of small satellites reduce mission failure risk because of their redundancy, there is much benefit to constellation-type missions such as CYGNSS. Other missions that also utilize existing signals of opportunity do not need to generate and send signals, like active remote sensing techniques, and are also therefore more cost effective.

This analysis quantified flood inundation dynamics due to Hurricanes Harvey and Irma by repurposing signals recorded by the CYGNSS constellation. These signals, opportunistically intercepted by CYGNSS, captured significant changes in inundation in both Texas and Cuba that took weeks to recede. Because the data provided by CYGNSS can map inundation at moderately high spatio-temporal resolution, they will be valuable in mapping flooding from future severe weather events.

## Methods

The primary observable in this paper are surface reflectivity (SR) observations, which were derived from Level 1 data recorded by CYGNSS. These observations were derived from delay-Doppler maps (DDMs) recorded by the CYGNSS receivers. DDMs represent the correlative power between the received, surface-reflected signal, and a replica signal stored within the receiver. The value of the peak cross-correlation of each DDM is related to surface characteristics at the specular reflection point of the GNSS signal—including the roughness of the surface and the surface dielectric constant^36,39,40^. At L-band, the surface dielectric constant is primarily a function of the moisture content, or wetness, of the surface, with wetter surfaces producing stronger reflections^18^.

The peak cross-correlation of each DDM is not only related to surface characteristics, but also affected by variables like the receiver antenna gain^39,40^. We corrected for these effects by assuming coherent surface reflections, which has been done in previous studies analyzing surface-reflected GNSS signals over land^36,41^.

The coherent component of scattered power is as follows:

1 $${P}_{rl}^{r}=\frac{{P}_{r}^{t}{G}^{t}}{4\pi {({R}_{ts}+{R}_{sr})}^{2}}\frac{{G}^{r}{\lambda }^{2}}{4\pi }{{\rm{\Gamma }}}_{rl}$$

where: ${P}_{r}^{t}$ is the transmitted RHCP power, ${G}^{t}$ is the gain of the transmitting antenna, ${R}_{ts}$ is the distance between the transmitter and the specular reflection point, ${R}_{sr}$ is the distance between the specular reflection point and the receiver, ${G}^{r}$ is the gain of the receiving antenna, *λ* is the GPS wavelength (0.19 m), and ${{\rm{\Gamma }}}_{rl}$ is the surface reflectivity.

In dB, this becomes:

2 $$10\,\mathrm{log}\,{P}_{rl}^{r}=10\,\mathrm{log}\,{P}_{r}^{t}+10\,\mathrm{log}\,{G}^{t}+10\,\mathrm{log}\,{G}^{r}+20\,\mathrm{log}\,\lambda +10\,\mathrm{log}\,{{\rm{\Gamma }}}_{rl}-20\,\mathrm{log}({R}_{ts}+{R}_{sr})-20\,\mathrm{log}(4\pi )$$

Solving for surface reflectivity, we have:

3 $$SR=10\,\mathrm{log}\,{{\rm{\Gamma }}}_{rl}=10\,\mathrm{log}\,{P}_{rl}^{r}-10\,\mathrm{log}\,{P}_{r}^{t}-10\,\mathrm{log}\,{G}^{t}-10\,\mathrm{log}\,{G}^{r}-20\,\mathrm{log}\,\lambda +20\,\mathrm{log}({R}_{ts}+{R}_{sr})+20\,\mathrm{log}(4\pi )$$

${P}_{rl}^{r}$ is calculated from the signal-to-noise ratio (SNR) observations of DDMs. SNR is the peak cross-correlation of the DDM, normalized to a noise floor, which is defined as the mean cross-correlation before leading edge of the reflection in the DDM. As other authors have pointed out^42^, SNR is related to, but is not exactly, ${P}_{rl}^{r}$, since SNR is affected by other factors like system noise levels and receiver instrument gain. It is for this reason that surface reflectivity (SR) is proportional to the CYGNSS observations:

4 $$SR\propto SNR-10\,\mathrm{log}\,{P}_{r}^{t}-10\,\mathrm{log}\,{G}^{t}-10\,\mathrm{log}\,{G}^{r}-20\,\mathrm{log}\,\lambda +20\,\mathrm{log}({R}_{ts}+{R}_{sr})+20\,\mathrm{log}(4\pi )$$

Because SNR is not in magnitude equal to ${P}_{rl}^{r}$, the observations and corrections made in Eq. 4 result in magnitudes greater than 70 dB. To produce values in a range that intuitively makes sense, we removed the mean of the bottom 5% of SR values (69.8731 dB) to produce the resulting SR observations shown in the text. Future attempts to retrieve the dielectric constant of the surface using an inverse model would require that SNR not be used as a corollary to ${P}_{rl}^{r}$.

In order to estimate the expansion of inundation extent due to the hurricanes, we employed a simple thresholding technique that has been used in previous studies with monostatic radar^14,24^, such that SR observations above a certain value are considered to come from inundated or partially inundated areas, and SR observations below the threshold do not come from inundated areas. As the CYGNSS data, and all L-band bistatic radar observations, are a new data type, there is relatively little guidance as to what this threshold value should be. Previous work has suggested that SR will increase as the percentage of open water in the sensing footprint increases, with a value of ~12 dB definitely indicating the presence of surface water, for an environment with low- to medium-vegetation density and typical roughness^43^. As we show in the text, a 12 dB threshold does produce inundation maps that outline water bodies, for at least the Texas coast and Cuba. Different thresholds would produce different estimates of inundated area, and this threshold would need to be tested in different environments if an operational flooding product were to be made from these data.

Although the time series examples shown in Fig. 2 are averaged with a daily time step, maps of inundated area in Figs 3 and 4 were derived using a compilation of observations over time, such that these maps indicate maximum inundation extent for the entire area. A compilation of observations was collected for these maps due to the pseudo-random sampling of GNSS-R satellites. Because both the transmitting and receiving sources are constantly moving, specular reflection points on the ground surface are pseudo-randomly distributed. For the maps of Texas in Fig. 3, we used observations collected between Jul 1 and Aug 20, 2017, (pre-hurricane image) and Aug 25 and Sep 15, 2017, (post-hurricane image). For the maps of Cuba in Fig. 4, we used observations collected between Jul 1 and Aug 20, 2017, (pre-hurricane image) and Sep 8 and Sep 30, 2017, (post-hurricane image).
